# In vivo biomechanical property detection of the ciliary muscle based on optical coherence tomography and digital image correlation technology

**DOI:** 10.1186/s40662-026-00498-w

**Published:** 2026-07-06

**Authors:** Jingsong Wang, Shenglong Luo, Yixin Li, Shenao Yi, Ning Xu, Tong Wang, Yang Gao, Ruyu Yan, Fan Lu, Junjie Wang, Yilei Shao

**Affiliations:** 1 National Clinical Research Center for Ocular Diseases, Eye Hospital, Wenzhou Medical University, No. 270 Xueyuan West Road, Wenzhou, 325027 Zhejiang China; 2 Zhejiang Key Laboratory of Ophthalmic Drug Discovery and Medical Device Research, Eye Hospital, Wenzhou Medical University, No. 270 Xueyuan West Road, Wenzhou, 325027 Zhejiang China; 3 Oujiang Laboratory, Zhejiang Lab for Regenerative Medicine, Vision and Brain Health, Wenzhou, 325101 China; 4 Department of Ophthalmology, The Third Hospital of Mianyang, Sichuan Mental Health Center, Mianyang, 621054 China

**Keywords:** Digital image correlation, Ciliary muscle, Biomechanics, Optical coherence tomography, Accommodation, Myopia

## Abstract

**Background:**

This study aimed to develop and validate a digital image correlation (DIC) methodology based on swept-source optical coherence tomography (SS-OCT) for quantifying in vivo ciliary muscle (CM) deformation and strain during accommodation.

**Methods:**

This study was comprised of three phases. First, the measurement performance was examined across two SS-OCT systems (CASIA2 and VG200D) and four scanning modes (R1, R8, R16, and R64) by comparing the DIC-derived displacements with the simulated ground-truth fields. Second, reliability was validated using a “cross-image” strategy, in which artificial deformations were applied to biological images to assess tracking accuracy under realistic speckle noise. Third, 23 subjects were recruited and divided into three groups: control group (CN, n = 7), high myopia group (HM, n = 7), and elderly group (n = 9). Macroscopic morphological parameters (anterior CM length change and maximum CM thickness change) and biomechanical parameters (e.g., equivalent strain) were extracted synchronously.

**Results:**

The R8 scanning mode in the CASIA2 device exhibited the most stable acquisition configuration. The robustness of the ncorr‑DIC algorithm was verified through the second-step experiment. Under the accommodative stimulus of − 5.0 D, the effective strain exhibited a highly significant graded attenuation among groups: CN (0.28 ± 0.06) > HM (0.20 ± 0.03) > Elderly (0.16 ± 0.05) (*P* = 0.002). Morphological changes were consistent with this finding, and CM thickening was significantly greater in the CN group than in the other two groups (*P* = 0.003). Combined analysis revealed that the mean effective strain was not only significantly positively correlated with macroscopic muscle thickening (*r* = 0.70, *P* < 0.001) but was also highly correlated with accommodation amplitude (*r* = 0.69, *P* < 0.001).

**Conclusion:**

DIC-based OCT analysis is feasible for assessing the biomechanical response of CM. By quantifying mechanical attenuation associated with HM and age-related decline, this method provides a new functional biomarker for investigating the mechanisms of accommodation dysfunction and guiding clinical interventions.

## Background

Ocular accommodation, a fundamental physiological process essential for achieving clear near vision [1], is primarily driven by ciliary muscle (CM) contraction and consequent crystalline lens reshaping. The biomechanical properties of the CM are critical initiators; abnormalities are closely associated with accommodation dysfunction and are significant in myopia progression and presbyopia development [2–5]. Optical coherence tomography (OCT) is widely used to evaluate CM contraction [6, 7]. However, these studies have mainly relied on morphological changes such as muscle thickening and forward movement. Although such image analysis provides anatomical structural information, effective methods for direct in vivo assessment of the displacement and strain distribution characteristics of different muscle groups remain limited [8].

Digital image correlation (DIC) may be used to analyze the strain distribution in CM under stress. As a non-contact, full-field optical measurement method, DIC tracks the displacement of random speckle patterns to calculate surface deformations with sub-pixel precision [9, 10], and was initially established for micron-scale strain analysis of engineering materials [11]. Recently, DIC has been increasingly used in biomedical applications, such as bone deformation, vascular wall strain, and corneal biomechanics [12–14]. OCT inherently generates speckle patterns that can serve as natural texture signatures for DIC tracking; however, achieving an adequate signal-to-noise ratio (SNR) to maintain stable speckles is critical for accurate correlation. Recent studies have successfully applied DIC algorithms to OCT images [15], demonstrating their potential for quantifying the displacement and strain distribution of CM in vivo. Within the framework of optical coherence elastography (OCE), tissue displacement and strain are typically quantified using phase-sensitive OCT systems that provide highly precise measurements [16, 17]. However, because commercial OCT devices often restrict access to raw phase data, this study utilized structural intensity images. Although tracking deformation via intensity-based DIC is inherently less precise than phase-sensitive OCE, it circumvents the need for custom-built hardware. This methodological tradeoff makes intensity-based tracking a broadly accessible alternative that facilitates clinical biomechanical assessments using standard commercial OCT systems [18].

Therefore, this study aimed to evaluate the feasibility of applying DIC to OCT imagery and to quantify the internal strain deformation of the CM during accommodation. This approach uses OCT speckles as a biomechanical tracking feature, treating them as encoded microstructural information that enables motion correlation and deformation extraction [19–21].

## Methods

### OCT imaging systems and settings

To evaluate the compatibility of different imaging protocols with DIC analysis, this study employed two clinically prevalent commercial swept-source optical coherence tomography (SS-OCT) systems that provide fast scanning. The VG200D (SVision Imaging Technology, China) operates at a central wavelength of 1050 nm with a scan speed of 200,000 A-scans/s and an axial resolution of 6.3 μm. The CASIA2 (Tomey Corporation, Japan) adopts a central wavelength of 1310 nm, achieving a scan speed of 50,000 A-scans/s and an axial resolution of 10 μm.

To determine the optimal image quality for speckle tracking, both devices were configured with four scanning modes based on the image-averaging repetitions (R1, R8, R16, and R64). The four mode parameters of VG200D were as follows: (1) AS Cornea, R1, 10 mm × 16.2 mm, 2509 × 4052 px, 0.01 s; (2) AS Sclera Contacts, R8, 18 mm × 16.2 mm, 4506 × 4097 px, 0.18 s; (3) AS Star, R16, 16 mm × 16.2 mm, 4002 × 4078 px, 0.32 s; and (4) AS Single-line, R64, 18 mm × 16.2 mm, 4503 × 4097 px, 1.44 s. In CASIA2, the scanning window was fixed at 12 mm × 11 mm with a pixel size of 1607 × 1473 px (acquisition times: R1, 0.03 s; R8, 0.21 s; R16, 0.42 s; R64, 1.66 s), and the imaging modes were varied solely by changing the number of averaging repetitions. Before imaging, participants were instructed to fixate steadily on the visual target. Therefore, fixation deviations and micro-eye movements were controlled to the greatest extent possible. The OCT images were acquired using the system's default internal calibration. Given that the DIC algorithm computes relative structural strain, a dimensionless ratio of displacement, rather than absolute anatomical depth, additional refractive index distortion correction was not applied because a constant refractive index assumption symmetrically affects pre- and post-accommodation scans. The images utilized were 2D cross-sectional B-scans and not en face images.

### Accommodative stimulation protocol

**Fig. 1:**
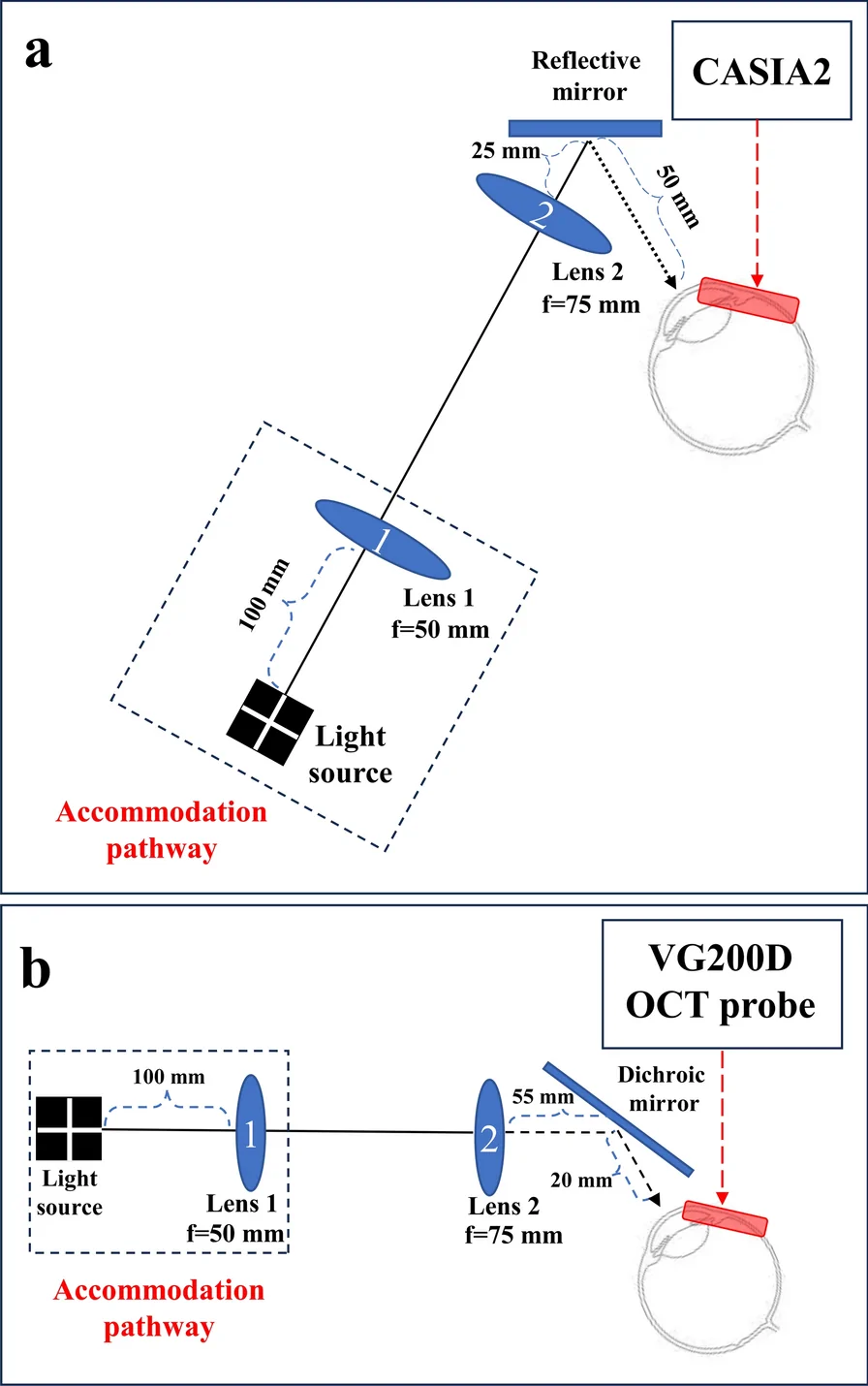
Experimental setup (a custom-built Badal optical system was combined with the optical coherence tomography [OCT]). **a** CASIA2-OCT combined with Badal optical system. **b** VG200D-OCT combined with Badal optical system. The distance between the visual target and Lens 1 is fixed at 100 mm, forming an image 100 mm behind the lens. When this image point coincides with the anterior focal point of Lens 2, collimated light is presented to the eye. A 1 diopter (D) change in vergence produced by this Badal system can be achieved by translating the visual target and Lens 1 forward together by 5.625 mm

To correct refractive errors and stimulate accommodation, a custom-built Badal optical system was combined with the OCT instruments [6, 22] (Fig. 1). The system comprised a white cross target on a black background (size: 0.4 logMAR) illuminated by an LED source. The 0.4 logMAR cross target was strategically selected to optimize the balance between inducing precise accommodation and accommodating myopic blur sensitivity [23]. The optical pathway included two convex lenses with focal lengths of 50 and 75 mm, along with reflective mirrors. Calibration confirmed that a 5.625-mm forward movement of the cross-target corresponded to 1 diopter (D) of accommodative demand.

### Experimental design and procedure

**Fig. 2:**
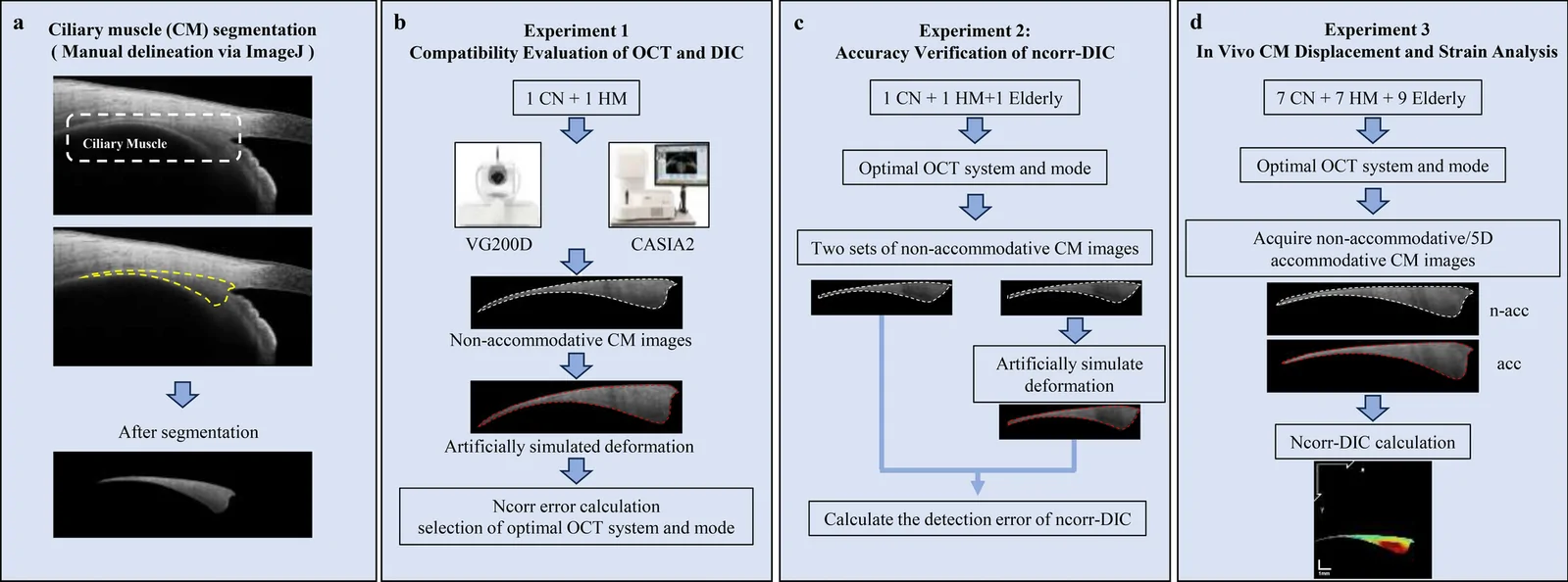
Experimental flow chart. **a** Schematic diagram of ciliary muscle (CM) segmentation. **b** Flow chart of Experiment 1. **c** Flow chart of Experiment 2. **d** Schematic diagram of Experiment 3. CN, control group; DIC, digital image correlation; HM, high myopia; OCT, optical coherence tomography

Three experiments were conducted to validate and apply the DIC method (Fig. 2). This study was approved by the Ethics Committee of Wenzhou Medical University (No. 2022-028-K-20-02) and was conducted according to the principles of the Declaration of Helsinki. All the participants provided written informed consent.

Experiment 1 determined the optimal OCT system and scanning mode for DIC analysis by benchmarking image performance against a known ground truth. CM images were acquired from two participants (one control and one with high myopia [HM]) in a non-accommodative state using all four scanning modes of the VG200D and CASIA2 systems. This phase was strictly a technical validation step to determine the theoretical hardware-algorithm compatibility and optimal scanning mode rather than a clinical population assessment. Different OCT modes yield different image qualities. Image averaging improves the SNR by suppressing incoherent random noise, which aids the DIC algorithm in recognizing stable structural speckles. However, prolonged averaging increases the susceptibility to physiological micromovements, causing speckle smearing. Thus, the optimal scanning mode was determined. To generate a reference for evaluation, complete CM images were segmented using the ImageJ software (National Institutes of Health, Bethesda, MD, USA) and subjected to artificial deformation simulations using MATLAB R2020a (MathWorks, Natick, MA, USA). In this study, only the segmented CM region was used for the DIC analysis. Direct processing of the original OCT images would substantially increase the computational cost and lead to an extremely low analysis efficiency. Using interpolation resampling methods, two levels of deformation were applied based on a previous accommodation database; “large” (horizontal compression: 200 μm, vertical stretch: 100 μm) and “small” deformation conditions (horizontal compression: 40 μm, vertical stretch: 10 μm). These physical values were converted into pixel-scale deformations based on the specific imaging dimensions of each OCT system (Table 1). Physical pixel size (μm/px) = (Actual length (mm) × 1000) / Number of pixels. Although the image pixel sizes of the VG200D varied across the different scanning modes, the actual physical sizes corresponding to each pixel were nearly identical. Thus, the physical size of the deformation was standardized. The open-source DIC algorithm ncorr [24] was used to analyze the simulated images. The identification errors (average) were calculated by comparing the ncorr-detected values with the artificially simulated inputs, and the system/mode configuration yielding the lowest error was selected for subsequent experiments. This experiment could only verify the detection accuracy of the algorithm for the simulated deformed images.

**Table 1:** Amount of artificially simulated ciliary muscle deformation

Deformation condition	Direction	CASIA2	VG200D
Large deformation	H	25 px	50 px
	V	15 px	25 px
Small deformation	H	5 px	10 px
	V	2 px	2.5 px

*H* = horizontal; *V* = vertical; *px* = pixel

Experiment 2 assessed the in vivo detection repeatability and anti-noise robustness of the DIC framework. The optimal imaging protocol determined in the first experiment was used to acquire CM images from three participants (Control [CN], HM, Elderly). For each participant, images were captured twice at 30-s interval in the non-accommodative state. One of the two raw images was randomly selected and digitally modified to obtain the same deformation magnitudes as those used in Experiment 1 (large and small). The ncorr-DIC algorithm is used to compute the displacement and strain differences between the modified and unaltered raw images. The accuracy of the algorithm was assessed by comparing the DIC-derived values with known simulated deformations. Using two independently acquired images with inherent noise variations, we simultaneously evaluated the precision and noise tolerance of the algorithm under real OCT speckle conditions.

**Fig. 3:**
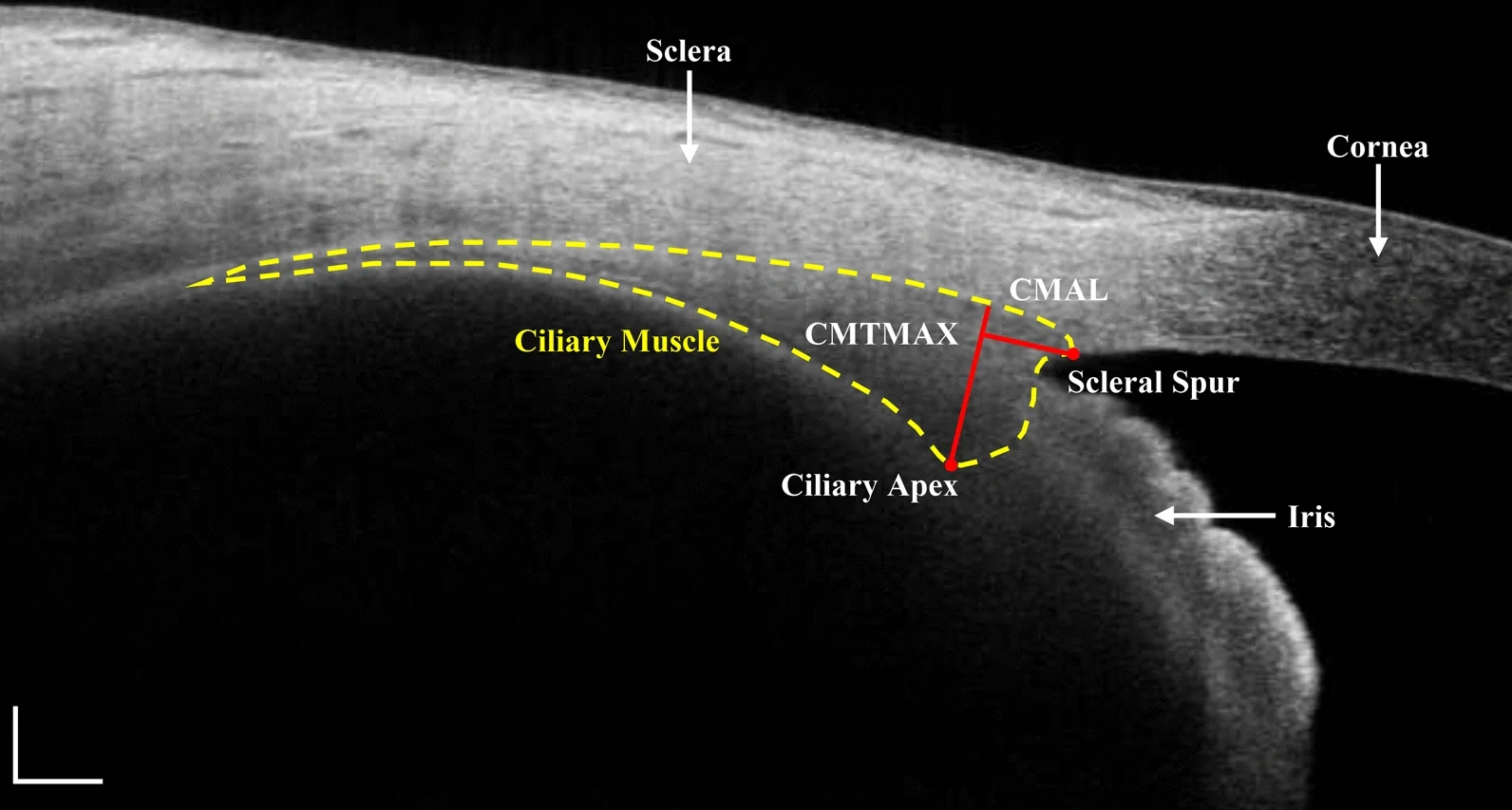
Schematic diagram of macroscopic morphological parameters of the ciliary muscle. CMTMAX, maximum thickness of the ciliary muscle; CMAL, anterior length of the ciliary muscle

Experiment 3 used a validated DIC protocol to evaluate the in vivo accommodative strain in CM. This phase included adults aged 20–50 years who were divided into three groups by refractive error and age: (1) CN group: adults aged 20–30 years; spherical equivalent (SE; spherical refraction plus half the cylindrical refraction) ranging from − 3.00 D to + 0.50 D; (2) HM group: adults aged 20–30 years; SE ≤  − 6.0 D or axial length (AL) ≥ 26.5 mm; (3) Elderly group: adults aged approximately 50 years; − 3.0 D ≤ SE ≤  + 0.50 D. All participants had corrected visual acuity of at least 20/20. The exclusion criteria were as follows: wearing contact lens within one week; history of ocular diseases, intraocular surgery, or related systemic diseases; and complications secondary to HM. All participants were imaged using an optimal OCT device and the scanning parameters were verified in Experiment 1. Images were acquired twice in the non-accommodative (n-acc) (baseline) state and once in the accommodative (acc) state. The ncorr-DIC method was used to calculate the displacement fields and strain distributions of the CM between the relaxed and accommodated states, reflecting its biomechanical properties. To further verify the feasibility of this method, macroscopic morphological changes in the CM were quantified. Taking the macroscopic morphological parameters as references, the biomechanical parameters (displacement and strain) derived from DIC were compared and analyzed. A custom semi-automated segmentation algorithm was applied to delineate three anatomical boundaries of the CM: air–conjunctiva, sclera–CM, and CM–ciliary body, following previously established method [5, 6, 25]. After segmentation and optical distortion correction, the maximum ciliary muscle thickness (CMTMAX) and ciliary muscle anterior length (CMAL) were quantified (Fig. 3). CMAL was defined as the linear distance from the scleral spur to the location of maximum CM thickness.

### Data processing and strain calculation

**Fig. 4:**
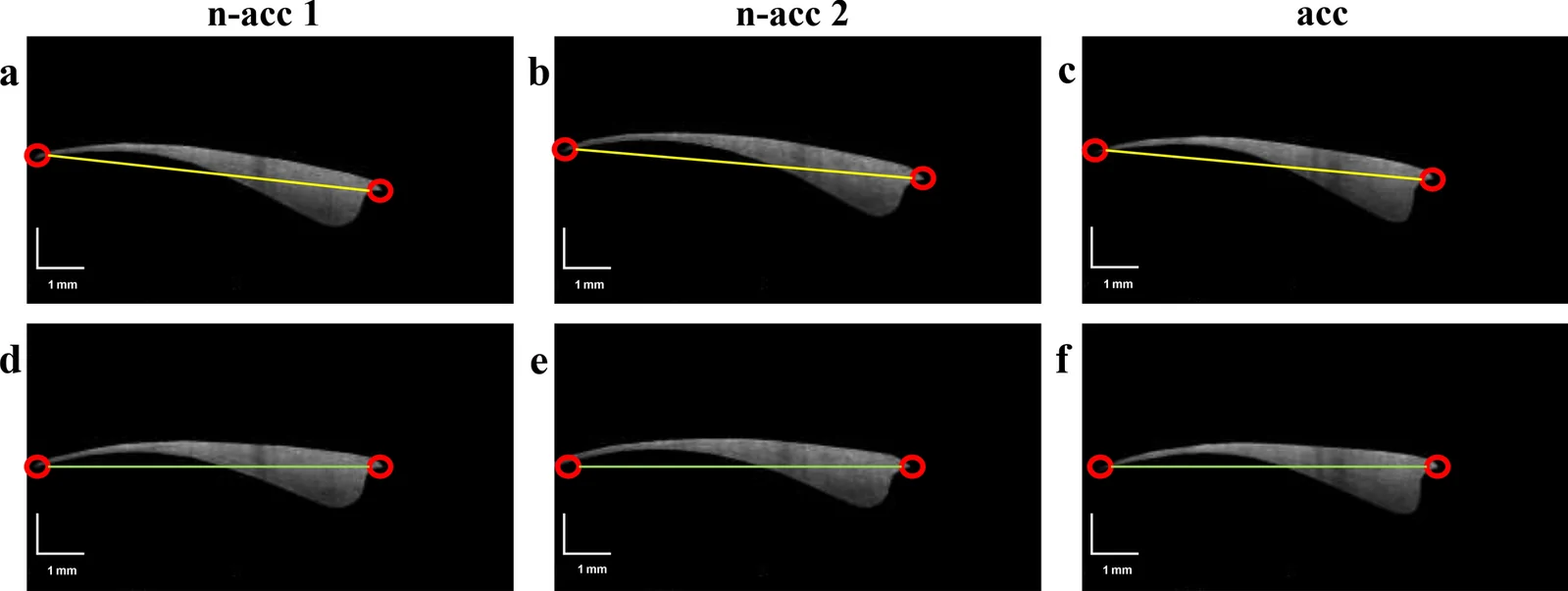
Schematic diagram of image registration. **a**, **b** Pre-registration n-acc CM (two acquisitions). **c** Pre-registration acc CM. **d**, **e** Post-registration n-acc CM (two acquisitions). **f** Post-registration acc CM. n-acc, non-accommodative state; acc, accommodative state; CM, ciliary muscle

To ensure that the measured displacement reflected internal tissue deformation rather than whole-eye movement, a rigorous image registration process was implemented before the DIC analysis. The DIC subset radius adopted in this study was 40 pixels with a step size of 1 pixel. All analyses were performed on raw OCT images without filter preprocessing. The registration mechanism ensured that the scleral spur [26] and the CM tail were positioned in the same horizontal position, with the scleral spur anchored to ensure identical coordinate positioning across both states (Fig. 4).

The original ncorr implementation was modified to incorporate automated region of interest (ROI) detection, enabling a more efficient and accurate localization of the CM. The ncorr analysis yielded five quantitative parameters: horizontal and vertical displacement vectors and horizontal normal (εₓₓ), shear (ε_xy_), and vertical normal strains (ε_yy_). The displacement field describes the overall movement of each point from its original position to the new position and is a vector with magnitude and direction. ε_xx_ and ε_yy_ measure “stretching” or “compression” in a single direction, whereas ε_xy_ reflects the sliding between internal layers [24]. Based on the 2D in-plane strain components obtained from DIC—ε_xx_, ε_yy_ and ε_xy_—the von Mises strain provides a single scalar value that summarizes the complex multidirectional strain state into a unified metric of total deformation. This metric was selected to represent the overall deformation intensity because it integrates these multidirectional strain components to provide a comprehensive assessment of tissue contractility while minimizing directional bias [27]. Consistent with previous anatomical studies [7, 28, 29], this analysis was restricted to the CM region within 3 mm of the scleral spur. Groupwise comparisons of the displacement and strain metrics were performed to characterize the accommodative biomechanical response and regional deformation behavior.

## Results

**Fig. 5:**
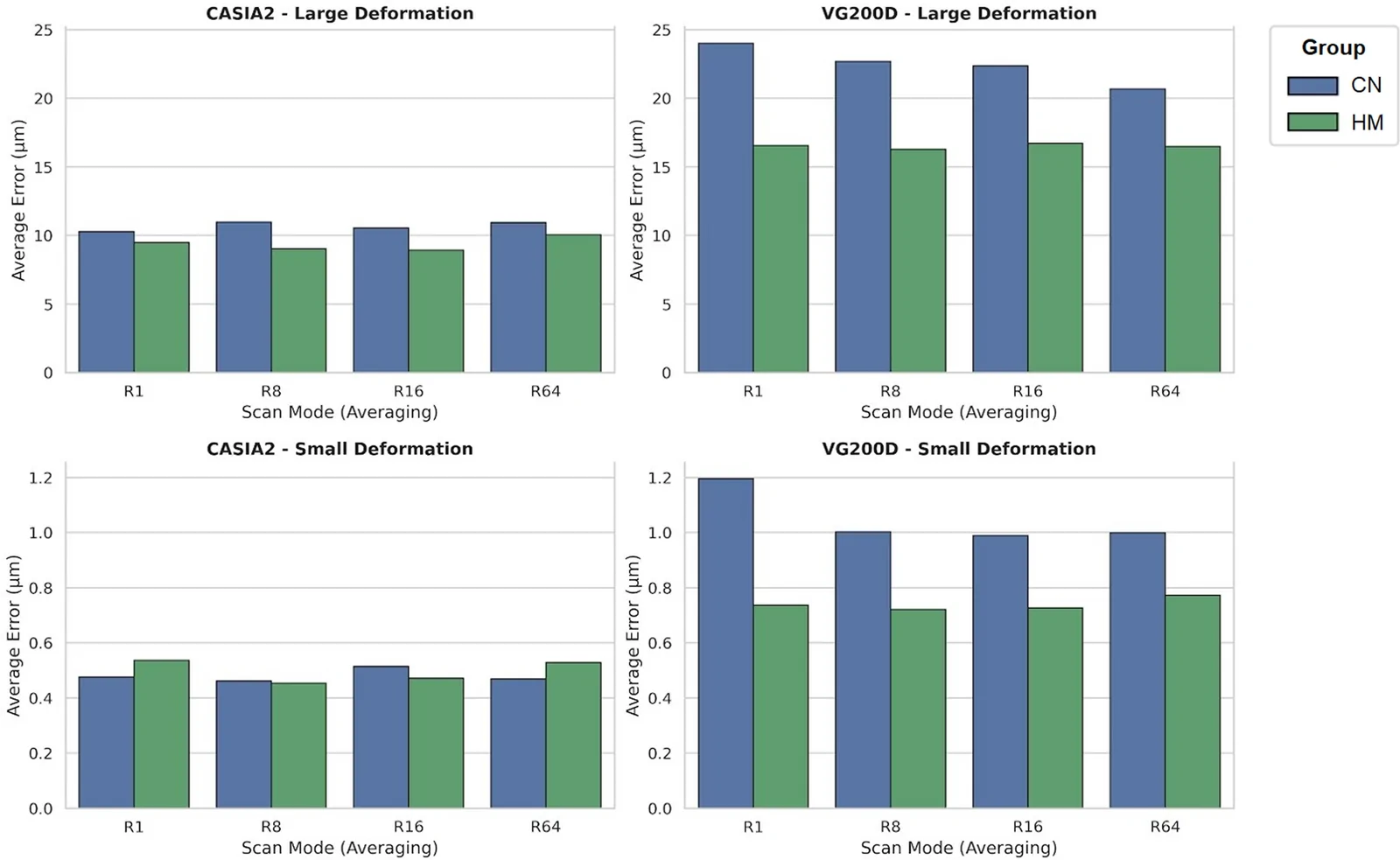
Comparison of average displacement tracking errors between CASIA2 and VG200D OCT systems. CN, control group; HM, high myopia

Experiment 1 used simulated deformation fields to evaluate the performance of the two SS-OCT systems via DIC analysis. The error evaluation revealed clear hardware-dependent differences: CASIA2 exhibited markedly lower error variability and superior detection stability compared to VG200D under large- and small-deformation conditions. Scan repetition affected performance, with the R8 mode of CASIA2 consistently producing relatively low identification error across most of the metrics (Fig. 5). Accordingly, CASIA2 in the R8 mode was selected as the optimal configuration and used for all subsequent in vivo experiments. Although the un-averaged R1 mode occasionally exhibited marginally lower errors for the CN group under specific metrics, it proved highly susceptible to random shot noise in the HM group, where tissue scattering and signal-to-noise ratios are inherently altered. Conversely, excessive averaging (R16 and R64) induced motion blur and over-smoothed the crucial high-frequency speckle patterns, degrading algorithmic tracking globally. Therefore, R8 was selected as the standardized consensus configuration, striking the optimal balance between shot-noise suppression and speckle preservation across diverse anatomical cohorts.

In Experiment 2, the analysis of cross-image deformation recovery showed that ncorr-DIC maintained a high detection accuracy despite inter-scan speckle decorrelation. As anticipated, compared with the perfectly idealized in silico simulations in Experiment 1, the tracking error in Experiment 2 exhibited a noticeable increase. This elevation in error provides direct evidence of natural speckle decorrelation between consecutive in vivo acquisitions of the CM, which inherently exerts a certain impact on the DIC pattern recognition process. Nevertheless, the average detection error for small deformations was approximately 20 μm. The error increased slightly under large deformation conditions, which may be attributed to individual differences and physiological fluctuations in accommodation (Table 2). These findings confirm that, in in vivo environments with inherent real speckles and physiological noise, the ncorr-DIC method can effectively identify tissue structures under diverse speckle backgrounds, possessing favorable anti-noise capability and reliable repeatability under realistic physiological conditions.

**Table 2:** Cross-image deformation tracking accuracy of the ncorr-DIC method under in vivo inter-scan speckle decorrelation

Group	Condition	Displacement direction	Average error (µm)
CN	Large deformation	Horizontal displacement	19.766
		Vertical displacement	13.388
	Small deformation	Horizontal displacement	16.945
		Vertical displacement	8.038
HM	Large deformation	Horizontal displacement	31.214
		Vertical displacement	14.638
	Small deformation	Horizontal displacement	21.965
		Vertical displacement	8.019
Elderly	Large deformation	Horizontal displacement	42.237
		Vertical displacement	27.386
	Small deformation	Horizontal displacement	15.629
		Vertical displacement	20.458

*CN* = control group; *HM* = high myopia; *Elderly* = elderly group

**Fig. 6:**
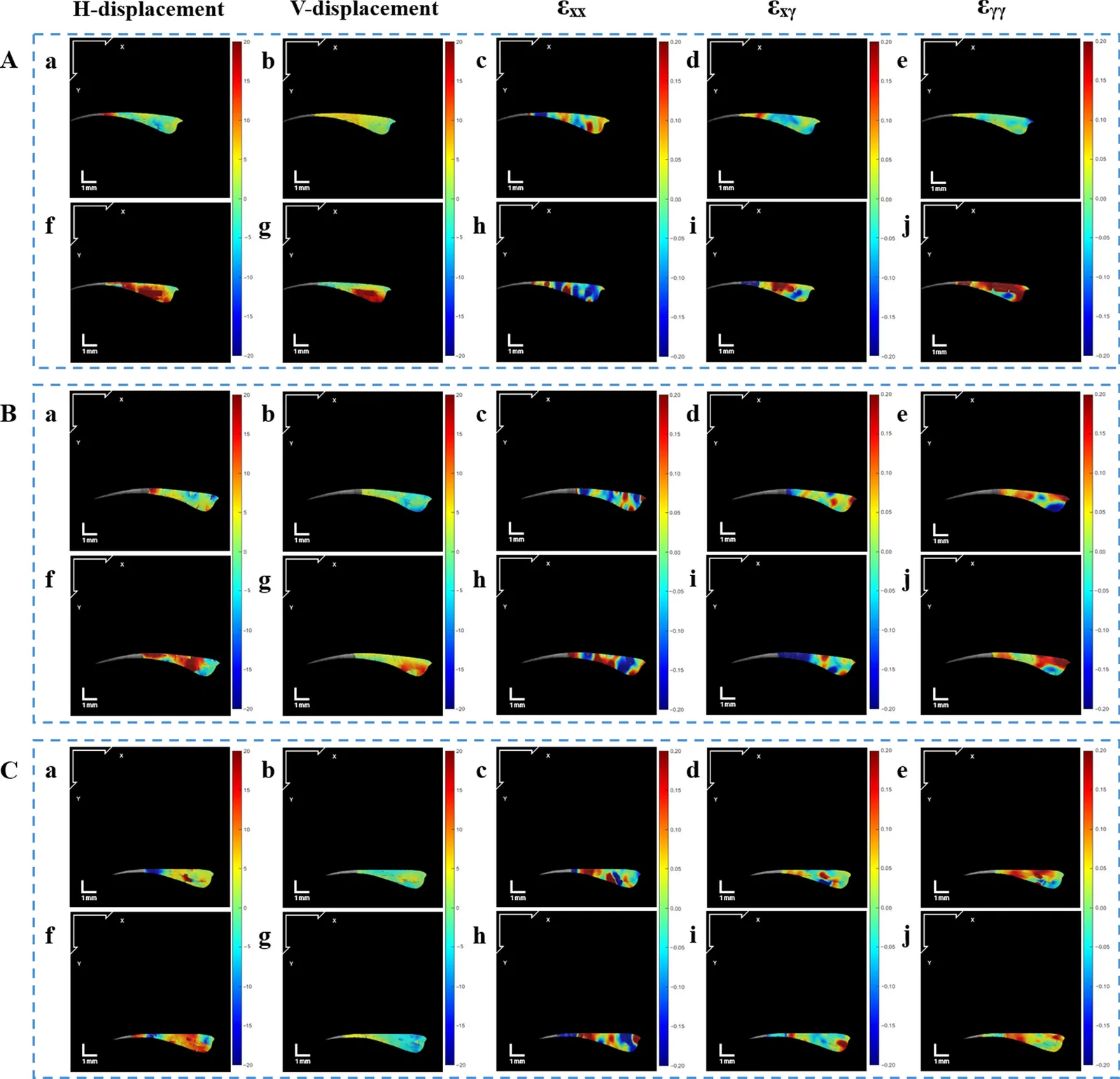
Schematic diagrams of ncorr-digital image correlation processing results for in vivo ciliary muscle deformation. A, Control group; B, High myopia group; C, Elderly group; **a–e** Test results of baseline repeatability (two acquisitions of n-acc ciliary muscle); **f–j** Test results under − 5.0 D accommodative stimulus; H, horizontal; V, vertical; εₓₓ, horizontal strains; ε_xy_, shear strains; ε_yy_, vertical strains; the unit of the displacement results is pixels; the strain results have no unit and can be understood as a percentage

**Fig. 7:**
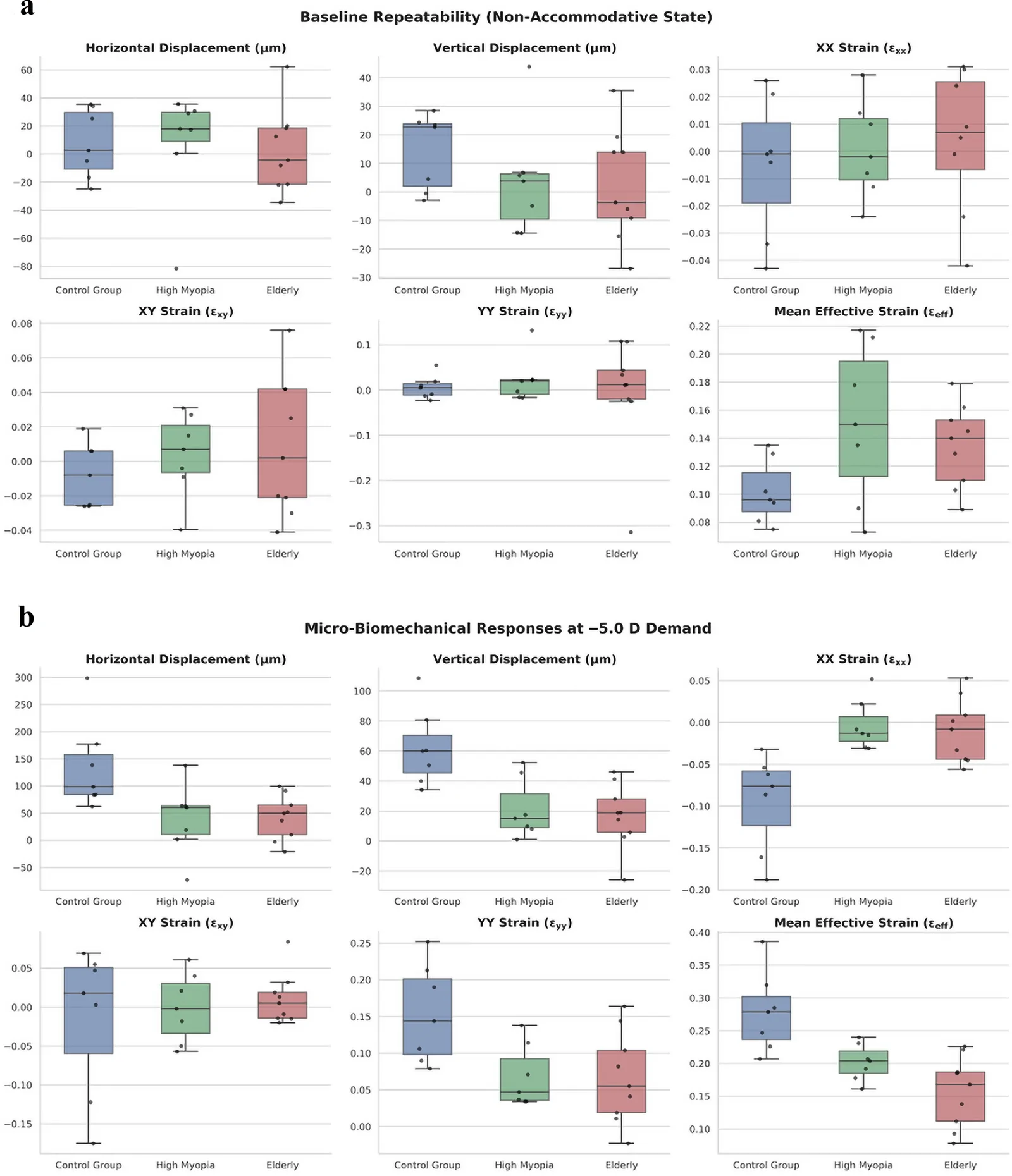
Results of ncorr-digital image correlation (ncorr-DIC) processing. **a** DIC processing results of the ciliary muscle under two non-accommodative states (i.e., baseline repeatability test); **b** DIC processing results of the ciliary muscle under − 5.0 D accommodative stimulus

In Experiment 3, 23 subjects were enrolled and their baseline characteristics are presented in Table 3. The active amplitude was measured using the negative lens method. At the microbiomechanical level, ncorr-DIC demonstrated a high tracking efficiency, with ROI recognition rates ranging from 81% to 98%. The ROI recognition rate represents the proportion of pixels successfully calculated using the ncorr algorithm relative to the entire CM ROI (i.e., the percentage of pixels identified by ncorr‑DIC relative to the total number of pixels within the entire CM region). Because the unrecognized areas were primarily concentrated in the posterior tail of the muscle, reliable data coverage was ensured within the target region, which was 3 mm posterior to the scleral spur. The ncorr-DIC processing schematic is shown in Fig. 6. Figure 6 is only used to show typical processing results of the ncorr-DIC method. Displacement and strain outcomes differ among individual subjects, and the overall statistical results are shown in Fig. 7. The horizontal displacement corresponds to the anterior–posterior (meridional) direction along the scleral inner wall. Positive values typically indicate anterior movement toward the scleral spur. Vertical displacement corresponds to the inward-outward (equatorial/centripetal) direction. Positive values indicate inward thickening of the crystalline lens and visual axis. Positive strain values indicate tissue tension (stretching), whereas negative strain values indicate tissue compression. According to the DIC analysis results, the mean values of displacement and strain obtained under the two non-accommodative conditions were extremely close to zero, with no significant differences among the three groups (Fig. 7). The baseline displacement and strain observed in the non-accommodative state may result from a combination of physiological micro-fluctuations (such as cardiopulmonary pulsation and resting tremor of the CM) and inherent measurement uncertainty of the algorithm. The baseline repeatability test indirectly verifies the tracking stability of the ncorr‑DIC method. Under the accommodative stimulus of − 5.0 D, the DIC-derived strain tensors revealed significant functional gradients among the cohorts (Fig. 7). The mean equivalent strain (ε_eff_), an aggregate scalar of principal contractions, demonstrated a highly significant stepwise attenuation: the CN group exhibited the highest intrinsic contractility (0.28 ± 0.06), followed by a significant decline in the HM group (0.20 ± 0.03), and the lowest contractility in the elderly group (0.16 ± 0.05) (overall *P* = 0.002). Analysis of directional kinematics indicated that physiological deformation was primarily driven by horizontal compressive strain (εₓₓ) and vertical tensile strain (ε_yy_), both of which were concomitantly diminished in the HM and elderly groups. Notably, the internal shear strain (ε_xy_) remained minimal and showed no statistically significant differences across any of the groups (*P* > 0.05).

**Table 3:** Basic characteristics of the three groups

Characteristics	CN (n = 7)	HM (n = 7)	Elderly (n = 9)	*P*
Sex (male/female)	2/5	1/6	3/6	–
Age (years)	25.29 ± 2.87	25.00 ± 0.82	52.44 ± 3.28	**< 0.001**
SE (D)	− 1.50 ± 1.12	− 7.46 ± 1.11	− 1.04 ± 1.70	**< 0.001**
AL (mm)	24.05 ± 1.07	26.48 ± 0.97	23.51 ± 1.44	**0.002**
IOP (mmHg)	15.44 ± 2.02	15.04 ± 2.91	14.59 ± 7.40	0.228
AMP (D)	9.14 ± 1.30	7.61 ± 0.59	2.86 ± 0.38	**< 0.001**

*P* values in bold indicate statistical significance

*CN* = control group; *HM* = high myopia; *Elderly* = elderly group; *SE* = spherical equivalent; *IOP* = intraocular pressure; *AMP* = accommodative amplitude. *P* values represent overall comparisons among the three groups

**Fig. 8:**
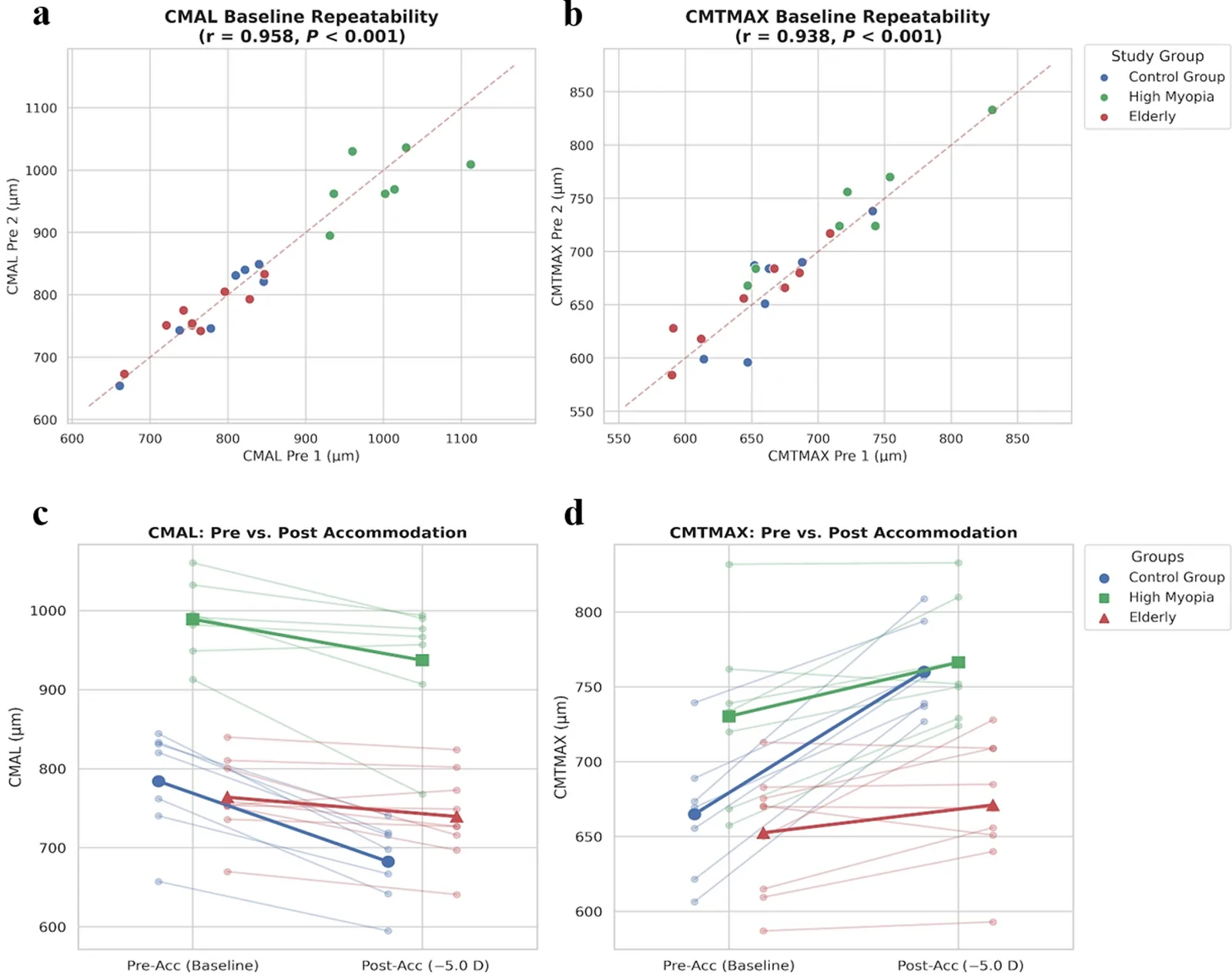
Results of macroscopic morphological changes. **a**, **b** Baseline measurement repeatability analysis (comparison of the ciliary muscle between two non-accommodative states). **c**, **d** Paired comparison before and after accommodation. CMAL, anterior length of the ciliary muscle; CMTMAX, maximum thickness of the ciliary muscle

**Fig. 9:**
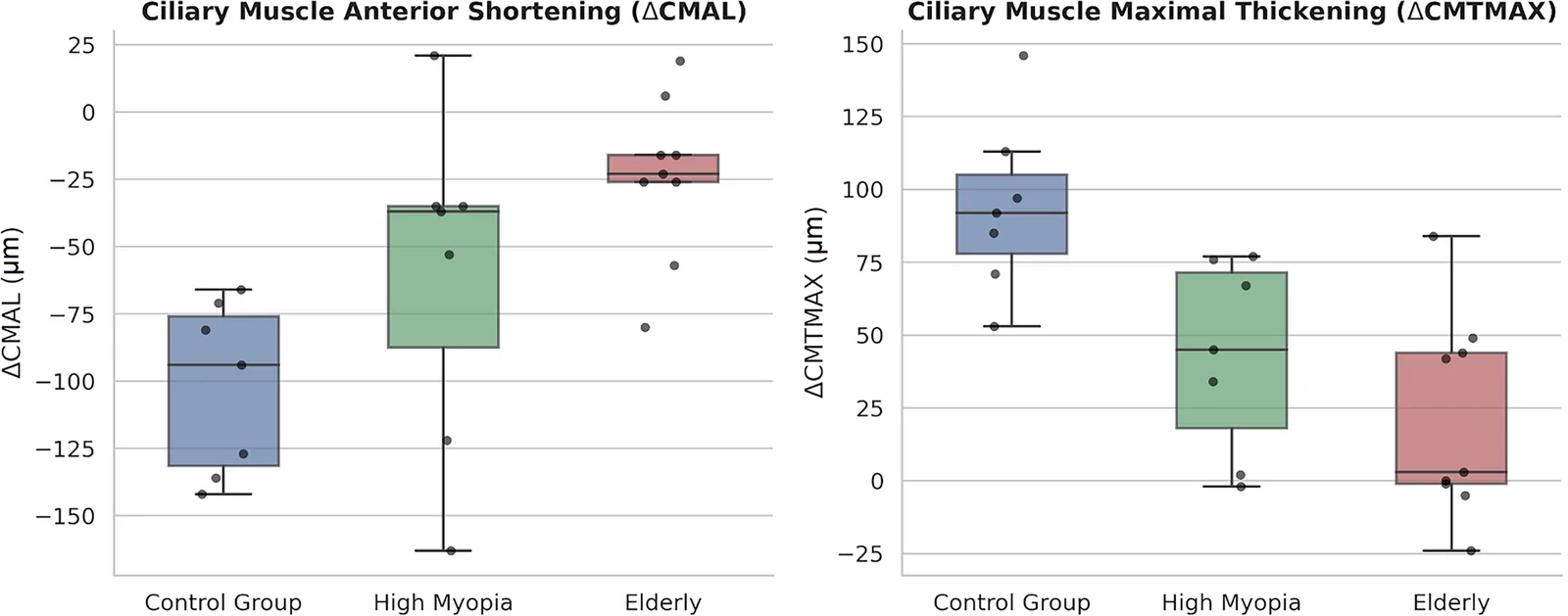
Comparison of anterior shortening (ΔCMAL) and maximal thickening (ΔCMTMAX) among groups. CMAL, anterior length of the ciliary muscle; CMTMAX, maximum thickness of the ciliary muscle

**Fig. 10:**
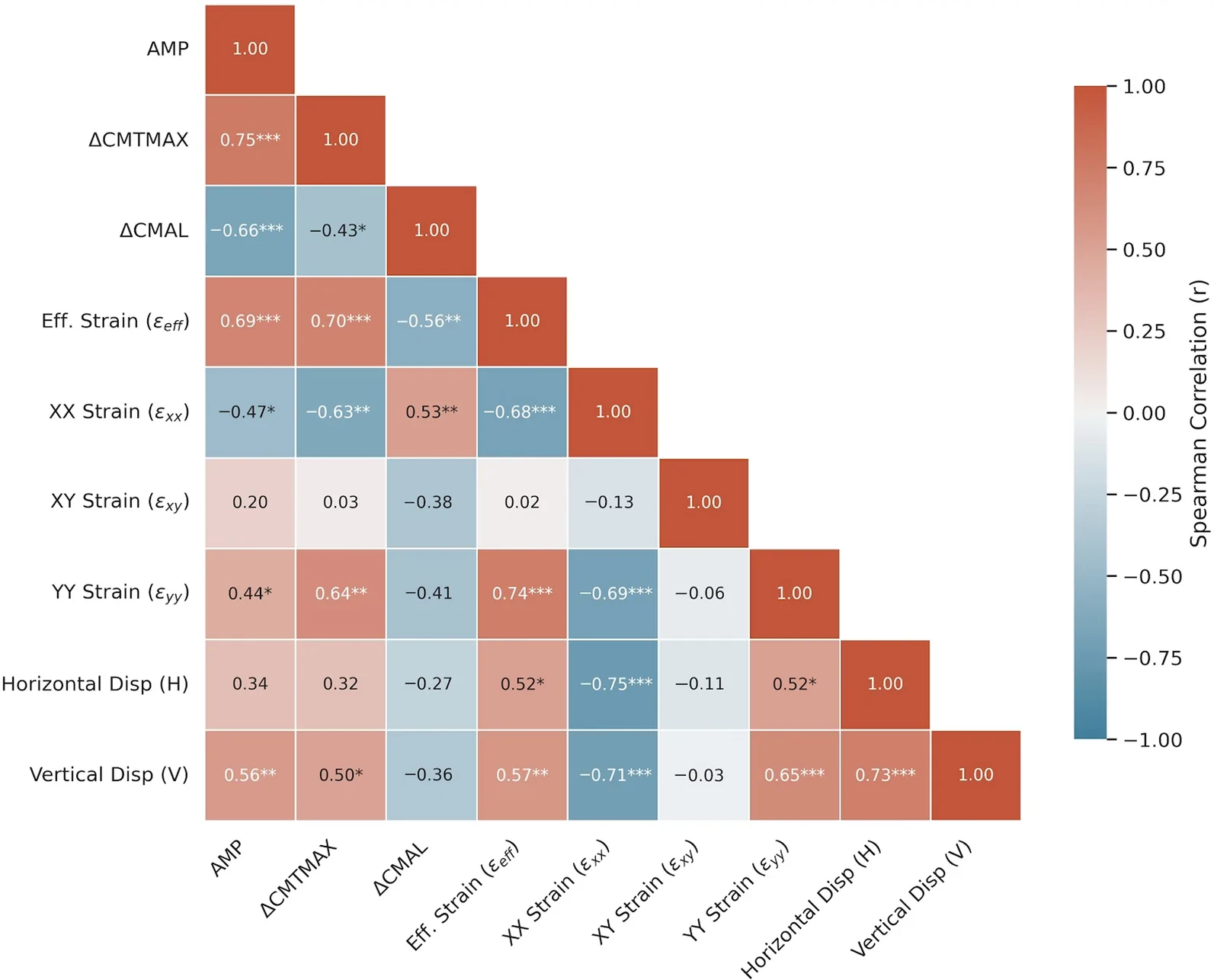
Correlation heatmap. AMP, accommodation amplitude; ΔCMAL, macroscopic maximal thickening; ΔCMTMAX, macroscopic anterior shortening; Eff. Strain, the mean equivalent strain; XX Strain, horizontal strain; XY Strain, shear strain; YY Strain, vertical strain; Disp, displacement; * *P* < 0.05, ** *P* < 0.01, *** *P* < 0.001

Morphological changes in CM biometry, including an increase in CMTM and a decrease in CMAL, were in accordance with those reported in previous studies [30, 31] (Fig. 8a and b). Upon accommodation, the CN group demonstrated robust morphological changes (Fig. 8c and d), characterized by a pronounced anterior shortening (ΔCMAL =  − 102.43 ± 31.99 µm) and a significant maximal thickening of the muscle belly (ΔCMTMAX = 93.86 ± 29.91 µm). Conversely, this accommodative thickening (increase in CMTMAX) was significantly reduced in both the HM and elderly groups compared to that in the CN cohort (overall Kruskal–Wallis, *P* = 0.003). The changes in CMAL and CMTMAX across the three groups are presented in Fig. 9. Finally, a combined correlation analysis was conducted to evaluate the interrelationships among microbiomechanics, macromorphology, and accommodative functions. The micro-level equivalent strain was significantly positive correlation with both the macroscopic muscle thickening (ΔCMTMAX, *r* = 0.70, *P* < 0.001) and the accommodation amplitude (*r* = 0.69, *P* < 0.001, Fig. 10).

## Discussion

This study demonstrated the first successful application of DIC to quantify the regional strain of CM in vivo. Traditional OCT analysis focuses on global morphological changes, such as thickness or anterior length (the straight-line distance from the scleral spur to the thickest portion of the CM) [7, 32], which reflect gross anatomical alterations, but cannot reveal internal deformation patterns. By contrast, the DIC-OCT approach moves beyond static boundary segmentation, enabling pixel-level structural speckle tracking to extract internal localized deformation fields. This establishes a new analytical pathway that moves beyond “shape change” to direct biomechanical readouts of accommodative function.

The reliability of applying DIC to the in vivo biomechanical detection of CM was underpinned by a systematic two-step validation of imaging and analytical protocols. In the first experiment, the imaging configuration was optimized to ensure stability of the speckle patterns required for DIC analysis. This comparison revealed that the CASIA2 system outperformed the VG200D, likely because its 1310-nm wavelength offers better tissue penetration and contrast for speckle tracking [33, 34]. The R8 scanning mode was identified as the optimal temporal balance. Image averaging fundamentally involves a tradeoff between SNR enhancement and spatial frequency preservation. While low averaging (R1) retains excessive random shot noise, prolonged high averaging (R64) acts as a spatial low-pass filter. It captures physiological micro-saccades over an extended acquisition window, thereby smearing the essential high-frequency speckle gradients necessary for precise cross-correlation.

With the second experiment, this study validated the algorithm’s accuracy using a “cross-image” strategy [9], applying artificial deformations to repeat biological scans rather than relying on pure simulations. This step was crucial to test the method’s robustness against realistic “speckle decorrelation” caused by physiological fluctuations and temporal noise between acquisitions. This study confirmed that despite the undeniable presence of natural speckle decorrelation and interscan optical noise, the optimized ncorr-DIC protocol maintained favorable detection accuracy.

Quantitatively, our in vivo macroscopic morphological findings served as a robust cross-validation of our imaging protocol. In the baseline cohort (CN), a − 5.0 D accommodative demand induced a mean ΔCMTMAX thickening of 93.86 ± 29.91 µm, demonstrating excellent quantitative concordance with established historical benchmarks [35–37]. Crucially, the micro-biomechanical strains extracted by our DIC framework align consistently with both the observed macroscopic morphological changes and the classical Helmholtz theory of accommodation. While these vectors intuitively align with hypotheses of myopia-induced mechanical restriction and presbyopic tissue stiffening, it is important to emphasize that the primary objective of this pilot study is methodological validation. Due to the relatively limited sample size, these physiological observations must be interpreted with caution. Rather than drawing definitive physiological conclusions, these results primarily serve as a proof-of-concept. The strong positive correlations between equivalent strain (ε_eff_) and both macroscopic thickening (*r* = 0.70) and clinical accommodative amplitude (*r* = 0.69) further confirm the reliability of the DIC method in reflecting true contractile work. Building on this validated consistency, DIC-based approaches have promising clinical implications. It provides a novel functional metric for evaluating CM biomechanics, which could be highly valuable for the early detection of presbyopia and objective assessment of anti-presbyopic interventions. Furthermore, it provides a quantitative tool for exploring the intricate involvement of accommodative functions in the onset and progression of myopia.

Despite these promising findings, the research is still at the methodological establishment stage, and therefore, has several limitations. First, the small sample size limits the generalizability of the results. Larger multicenter cohorts are necessary to confirm population-level consistency, and future large-scale studies must establish an absolute normative database using strictly emmetropic cohorts. Second, the analysis focused solely on a − 5.0 D stimulus and lacked a dose–response analysis under different amplitudes or a dynamic time-series analysis during the accommodation process [38]. Future research should explore the quantitative relationship between the strain and stimulus intensity to establish complete mechanical response models. Third, maintaining the OCT speckle stability remains challenging [39]. Although the imaging parameters were optimized, noise differences between acquisitions still caused instability in the displacement and strain results, suggesting that the data represented the overall CM information. Future iterations could integrate artificial intelligence algorithms to develop more robust speckle-tracking methods [40] and enhance distinguishability among muscle fiber groups. The reliance on 2D cross-sectional B-scans to evaluate an inherent 3D sphincter contraction represents an intrinsic physical constraint. Any out-of-plane (elevational) tissue motion orthogonal to the imaging plane inevitably induces speckle decorrelation, thereby introducing theoretical limits to 2D strain estimation. Fully resolving the true 3D strain tensor requires future transitions to volumetric SS-OCT and 3D Digital Volume Correlation (DVC) algorithms. Finally, anterior segment OCT is subject to geometric optical distortions. While our relative comparative design mitigates absolute errors, future iterations should incorporate comprehensive ray-tracing corrections.

## Conclusion

This study established and validated a novel non-invasive framework for quantifying in vivo CM biomechanics by integrating DIC with SS-OCT. Through systematic hardware optimization and rigorous error analysis, we confirmed the reliability of this method for tracking tissue deformation under physiological conditions. This preliminary application successfully captured distinct strain gradients across the participants, marking a critical transition from static morphological observations to dynamic functional assessments. As a proof-of-concept, this study demonstrated that OCT-based DIC is a reliable tool that provides a quantitative foundation for future large-scale investigations of the biomechanical mechanisms of myopia progression and presbyopia.

## Data Availability

The datasets generated and analyzed during this study are available from the corresponding author upon reasonable request.

## Abbreviations

CM: Ciliary muscle
OCT: Optical coherence tomography
DIC: Digital image correlation
CN: Control group
HM: High myopia
CMAL: Ciliary muscle anterior length
CMTMAX: Maximum ciliary muscle thickness
